# Supplementary material for: Patients’ experiences and perceptions of Guillain-Barré syndrome: A systematic review and meta-synthesis of qualitative research
Source: PLoS One. 2021 Feb 3;16(2):e0245826. doi: 10.1371/journal.pone.0245826 (PMC7857557; doi:10.1371/journal.pone.0245826)
Supplement: S2 File — (PDF) [file pone.0245826.s002.pdf]

Patient experiences and perceptions of Guillain-Barre Syndrome and associated inflammatory neuropathies following discharge from hospital

Ffion Curtis, Despina Laparidou, Joseph Akanuwe, Victoria Ellis-Vowles, Jennifer Jackson, Mahendra Senevirathna, Igor Menezes, Tim Hodgson, Aloysius Niroshan Siriwardena

Citation

Ffion Curtis, Despina Laparidou, Joseph Akanuwe, Victoria Ellis-Vowles, Jennifer Jackson, Mahendra Senevirathna, Igor Menezes, Tim Hodgson, Aloysius Niroshan Siriwardena. Patient experiences and perceptions of Guillain-Barre Syndrome and associated inflammatory neuropathies following discharge from hospital. PROSPERO 2019 CRD42019122199 Available from: http://www.crd.york.ac.uk/PROSPERO/display_record.php?ID=CRD42019122199

Review question

What are patients' experiences and perceptions following discharge from hospital and during recovery of Guillain-Barre Syndrome or associated inflammatory neuropathies?

Searches

The following databases will be searched: MEDLINE, CINAHL, EMBASE, PsycINFO, EBSCOhost, Academic Search Complete, Humanities International Index, PsycARTICLES, Sociological Abstracts, AMED, Web of Science, the Cochrane Library, Joanna Briggs Institute and PROSPERO. All databases will be searched from inception. Database searches will be supplemented with internet searches (i.e. Google Scholar), and forward and backward citation tracking from the included studies and review articles.

Types of study to be included

Qualitative, individual or group interview, focus group, ethnographic.

Condition or domain being studied

Patients' experiences, perceptions of Guillain-Barre Syndrome (Acute Inflammatory Demyelinating Polyneuropathy) or an associated inflammatory neuropathy such as Miller-Fisher Syndrome or Chronic Inflammatory Demyelinating Polyneuropathy.

Participants/population

Studies will be included if they involve qualitative analysis, are published in the English language, and are published between January 2000 and June 2018 to ensure relevance to the present day. Primary studies will report experiences from adults (aged 18 and over) with, or recovering from Guillain-Barre Syndrome (Acute Inflammatory Demyelinating Polyneuropathy) or a related condition such as Miller Fisher Syndrome or Chronic Inflammatory Demyelinating Polyneuropathy.

Intervention(s), exposure(s)

A previous diagnosis of Guillain Barre Syndrome (Acute Inflammatory Demyelinating Polyneuropathy) or an associated inflammatory neuropathy such as Miller Fisher Syndrome or Chronic Inflammatory Demyelinating Polyneuropathy.

Comparator(s)/control

No comparator

Context

Participants living in the United Kingdom or Republic of Ireland with a previous diagnosis of Guillain Barre Syndrome or an associated inflammatory neuropathy.

Main outcome(s)

Qualitative data: the experiences and views of people post discharge from hospital and returning to the

community after Guillain Barre Syndrome or associated inflammatory neuropathy.

Timing and effect measures

Additional outcome(s)

None

Timing and effect measures

Data extraction (selection and coding)

Titles and/or abstracts of studies retrieved using the search strategy and those from additional sources will be screened independently by two reviewers to identify studies that potentially meet the inclusion criteria outlined above. The full text of these potentially eligible studies will be retrieved and independently assessed for eligibility by two review team members. Any disagreement between them over the eligibility of particular studies will be resolved through discussion with a third reviewer.
A standardised, pre-piloted form will be used to extract data from the included studies for assessment of quality and data synthesis. Extracted information will included: study details (title, authors, date), methods (aims, objectives, research questions, study design, setting, data collection methods, outcomes, data analysis, context in terms of findings and relevant theory), and participants (demographics, inclusion/exclusion criteria, method of recruitment, sample selection and sample size). Two reviewer authors will extract data independently, discrepancies will be identified and resolved through discussion (with third reviewer where necessary). Missing data will be requested from study authors.

Risk of bias (quality) assessment

The CASP Qualitative Checklist (2017) will be used to assess risk of bias in included studies, however, quality will not be a sole reason for exclusion. A discussion on the quality of the articles will be included in the final review. Studies will be appraised by two reviewers independently with discrepancies being resolved by a third reviewer.

Strategy for data synthesis

Thematic synthesis, as described by Thomas and Harden (2008 will be used to synthesise qualitative data. This will involve three stages: 1) free line-by-line coding of the findings from primary studies; 2) the organisation of these 'free codes' into related areas to construct 'descriptive' themes; and 3) the development of 'analytical' themes.

Analysis of subgroups or subsets

None

Contact details for further information

Ffion Curtis
fcurtis@lincoln.ac.uk

Organisational affiliation of the review

University of Lincoln

Review team members and their organisational affiliations

Dr Ffion Curtis. University of Lincoln, UK
Despina Laparidou. University of Lincoln, UK
Dr Joseph Akanuwe. University of Lincoln, UK
Mrs Victoria Ellis-Vowles. University of Lincoln, UK
Dr Jennifer Jackson. University of Lincoln, UK
Dr Mahendra Senevirathna. University of Lincoln, UK
Dr Igor Menezes. University of Lincoln, UK
Professor Tim Hodgson. University of LIncoln, UK
Professor Aloysius Niroshan Siriwardena. University of Lincoln

Anticipated or actual start date

01 November 2018

Anticipated completion date
01 September 2019

Funding sources/sponsors
GAIN: Guillain-Barre and associated Inflammatory Neuropathies Charity

Conflicts of interest

Language
English

Country
England

Stage of review
Review_Ongoing

Subject index terms status
Subject indexing assigned by CRD

Subject index terms
Guillain-Barre Syndrome; Hospitals; Humans; Patient Discharge; Peripheral Nervous System Diseases

Date of registration in PROSPERO
23 January 2019

Date of publication of this version
23 January 2019

Details of any existing review of the same topic by the same authors

Stage of review at time of this submission

Stage	Started	Completed
Preliminary searches	Yes	Yes
Piloting of the study selection process	Yes	Yes
Formal screening of search results against eligibility criteria	Yes	Yes
Data extraction	Yes	No
Risk of bias (quality) assessment	No	No
Data analysis	Yes	No

Versions
23 January 2019

PROSPERO
This information has been provided by the named contact for this review. CRD has accepted this information in good faith and registered the review in PROSPERO. CRD bears no responsibility or liability for the content of this registration record, any associated files or external websites.
