# Supplementary material for: Patients’ experiences and perceptions of Guillain-Barré syndrome: A systematic review and meta-synthesis of qualitative research
Source: PLoS One. 2021 Feb 3;16(2):e0245826. doi: 10.1371/journal.pone.0245826 (PMC7857557; doi:10.1371/journal.pone.0245826)
Supplement: Attachment — Submitted filename: Response to reviewers.docx [file pone.0245826.s003.docx]

| **Comment** | **Our response** (page numbers refer to the Clean Manuscript) |
| --- | --- |
| **Editor**: Please ensure that your manuscript meets PLOS ONE's style requirements, including those for file naming. | We have made relevant changes throughout the manuscript. |
| We note that your search concludes in November 2018.  It is not clear why studies published in the last 12 months were not included for consideration.  Please provide a rationale for the search window, or update the search to include studies from the last 12 months. | Thank you. We have updated the searches (May 2020) and changed accordingly the information provided in the abstract, methods and results sections. No new studies were found. |
| The main outcome of the study should be summarize as last sentence of the introduction. | We have now added this sentence at the end of the Introduction (p. 7): “The findings of this review will provide insights into the patient journey, which could be useful in informing patient care and support services.” |
| The subchapter “Theoretical perspective” should be shortened by 50%, the introduction section is lengthily and not focused on the primary end point. | We have shortened this section considerably. |
| The Table should be reported at the end of the manuscript, and cited in the text. | Thank you, we have now moved all tables at the end of the Manuscript. |
| The Result section is composed by 20 pages. The Section is too lengthily. Please report only the results statistically significant in the text, the all results should be reported only in the Table. | Thank you, we have shortened this section, by including a short summary of findings in the main text and placing all quotes within Table 4 (p. 31), as suggested by the reviewer. |
| The first sentence of the Discussion section should summarize the main results of the study. | We have added the following sentence, summarising our main results as the second sentence of the discussion section (p. 20)- we hope that will be acceptable: “The findings of the review have shown that the participants’ experiences of recovery varied significantly, with many still experiencing residual (physical, psychological and social) difficulties even years after their discharge from hospitals.” |
| Also the Discussion section is long and lengthily. Please shortened by 30 to 40% and focused it on the primary end points of the study. | Thank you, we have now shortened the Discussion considerably |
| **Reviewer 1**: I've read with great interested this work by Siriwardena et al. dedicated to patients’ perceptions of Guillain-Barré syndrome. Undoubtedly, the data presented in this article are of great interest for specialists working with such patients. However, in my opinion, this article is extremely overloaded with data, which makes it really huge and looks like small book or thesis but not journal article. For instance, discussion section essentially repeats, in an abbreviated version, most of the theses set out in the results. | Thank you. We have now shortened both the Results & Discussion sections considerably. |
| The design of the study is not completely looks optimal to me, because it is not clear what new information this meta-synthesis provides us, compared to the five qualitative studies that were included in the analysis. What has changed in our understanding of the problem after this work? | This was an omission on our part, thank you. We have now added this in the Discussion section, under the strengths and limitations (p. 23): “This review has brought together papers discussing different aspects of people’s experience (such as during an acute episode [20] or the initial phase of GBS [17], the recovery phase [16, 18], and their experiences returning to work [19]) and has synthesised them for the first time into a comprehensive overview of the illness and recovery journey of people diagnosed with GBS.” |
| It is also not entirely clear why the authors excluded quantitative works, because, at least, discussion sections of them could also contain some useful information. | Thank you, we have provided the following explanation for our decision (p. 8): “Qualitative studies published outside these dates or in other languages were excluded. Quantitative studies were also not eligible for inclusion, since we were interested in patients’ lived experience of GBS and CIDP and we wanted to include in depth accounts of their experiences (preferably expressed in their own words, i.e. by using quotes).” |
| **Reviewer 2**: The authors confuse Reporting Standards with Standards of Conduct. Following good reporting standards does not ensure a good review and so it would be good to only cite reporting standards to affirm what they are intended for. | Thank you. We agree, as that was never our intention, and have now changed the sentence. It now reads: “The study followed a rigorous pre-specified protocol (registered with PROSPERO), which ensured that the review process was transparent and replicable.” |
| Certain points betray gravitation towards a quantitative paradigm e.g. discussion of "risk of bias", "blinding to authors", numbers of participants supporting a finding and numbers of studies required in a qualitative synthesis. | Thank you, we agree. We have changed “risk of bias” into “assessing quality”. We have also removed the sentence regarding blinding to authors, and have re-phrased sentences throughout the results section to show that we report experiences that were important to the participants (regardless of the number of participants supporting a finding). We have also removed any mention of the number of studies required in a qualitative synthesis, as we agree that similar arguments are more relevant to a quantitative review. |
| Particularly missing (to address some of these) is a "reflexive statement" see example below given from this same target journal. This will also address potential influence of the charity as funder (see below). | Thank you, we agree and have now added a short section at the end of the Methods (p. 10). |
| The authors present a "bricolage" of findings in the Results. Not only does it engage with the conventions of quantitative research (e.g. "most" participants and "many" participants) but it attributes specific individual statements to groups of individuals misrepresenting an individually worded comment as if an agreed common experience. The whole Results section needs rewriting framing findings as overall statements followed by illustrative verbatim extracts (judiciously selected). In practical terms the authors should look at each verbatim extract and ask: does this capture a common experience? Can I frame these related extracts in my own words as generalities, or is this a unique insight? Does this wording express the experience better than I can myself? At the moment these results are knitted together from the reviewers, the source authors and the verbatim extracts of participants. | We have rephrased sentences throughout the results section to better illustrate that we have described common experiences (common across participants within the same paper, but also common across different papers). |
| Although studies are not excluded on the basis of quality it would be useful to at least comment on CASP questions that the collective set of five studies performed less well against (typically reflexivity performs less well). | Thank you - we have now expanded the “Quality assessment of studies” section under the Results (p. 13) to include this sentence: “It should be noted here that three [16, 17, 20] of the five studies performed less well on the reflexivity question (CASP 06) and one study [17] performed less well on four CASP questions, since not enough relevant information was reported in the papers.” |
| “Due to the rarity of this condition there is limited evidence exploring the experiences of individuals who have had GBS.” This sentence perpetrates a common fallacy i.e. that the prevalence of research is linked to prevalence of a condition. In many cases the reverse is true (and this has been particularly recorded for neurological disorders) ie. That rare conditions are more attractive to research than common ones. This is partly attributable to the fact that journals are part scientific documentation and part journalism, partly to the “politics” of research funding and partly to other factors. | We have changed this sentence into (p. 4): “Currently there is limited evidence exploring the experiences of individuals who have had GBS.” |
| “CIDP” – this appears in the Review question without being written in full. The review question should have both conditions in full with respective abbreviations in brackets. (Otherwise CIDP is used twice BEFORE being written in full). It is not appropriate to use unexplained abbreviations in the review question. | We have corrected this (p. 7), thank you. |
| “January 2000 and November 2018, to ensure relevance to the present day,” This reads as a weak justification for date limit (cynically, I would say that you have probably selected an easily memorable but topically meaningless start date!). | We have now provided a more detailed explanation for our choice of dates (p. 7): “Healthcare services (including the treatment, care and support they provide) have changed considerably in the last 20 years and consequently so has patient experience of care. To ensure that the reviewed patient experiences are relevant to the present day and can inform improvements in current practice, only studies published between January 2000 and May 2020 were eligible for inclusion. In addition, only peer reviewed studies, written in English were considered for eligibility.” |
| “Joanna Briggs Institute” – It is unclear what this Data Source is. | We have changed this (p. 8) to “Joanna Briggs Institute Evidence-Based Practice Database” |
| “The search terms were entered in all possible combinations using Boolean operators and truncation, wherever deemed necessary” Not true! They would have been kept in sets of related concepts i.e. the GBS concept, the qualitative research concept. Not ALL possible combinations. Search terms should also best be presented as two sets for greater clarity. E.g. “(i) Guillain-Barré syndrome (GBS), chronic inflammatory demyelinating polyneuropathy (CIDP), acute inflammatory demyelinating polyneuropathy (AIDP) combined with (ii) qualitative research, interview, focus group, experiences, perceptions, attitudes, and views”. | We have made relevant changes (p. 8): “The search strategy used in all the above databases included a combination of two sets of keywords and related terms: 1) Guillain-Barré syndrome (GBS), chronic inflammatory demyelinating polyneuropathy (CIDP), acute inflammatory demyelinating polyneuropathy (AIDP); combined with 2) qualitative research, interview, focus group, experiences, perceptions, attitudes, and views. The search terms were entered using Boolean operators and truncation, wherever deemed necessary.” |
| “All studies were reviewed and screened by three reviewers” This is very unclear. Does this mean that “all references were reviewed and screened”. In a review “studies” don’t become “studies” until they are included. Before that they are first “references” and then “(full text) papers” | We have replaced the word “studies” with the word “references”. |
| “Qualitative Checklist [15] was used to assess risk of bias in included studies of this review” – The CASP checklist is not validated for “risk of bias”. Indeed what “risk of bias” means in a qualitative sense is epistemologically unclear anyway. Describe it only as “quality assessment”. | We have made relevant changes under the Abstract and Methods sections |
| “Quality, however, was not a criterion for exclusion of a study” This is a sound decision but requires a sentence or clause of explanation e.g. “given that the intent was interpretative….” Or something similar. Also, being accurate “quality” is not a criterion. “Poor quality” would be. | We have now changed this sentence (p. 10): “Low quality, however, was not a criterion for exclusion of a study, since we were interested in the synthesis and interpretation of all relevant and sufficiently rich data” |
| “Blinding to the names of the authors, journals, and results of each study was not possible, since the same authors performed the screening and data extraction of the studies to be included in the meta-synthesis.” You don’t typically see this spelled out, particularly as you don’t explain why this could be an issue. In my view this is a very quantitative way of handling a legitimate concern. In a QUALITATIVE synthesis like this I would expect instead a reflexivity statement that talks about all the authors’ positionality to the data. | Thank you. We agree and have removed this sentence. We have also added a reflexivity section (p. 10) as per your very useful suggestion. |
| “Initially, participants from most included studies” Avoid this quantitative reporting, especially when you then attribute multiple studies to a single verbatim extract as in this example. This should be reported as:  “Participants variously described initial experiences associated with their first awareness of their condition: “strange or odd sensations or peculiar feelings”, such as tingling in their feet or having difficulty opening the lid of orange juice containers, etc.  Similarly with “Most participants, “tried to ignore the strangeness of their bodies” whereas”. Especially be aware that “most participants” or “many participants” from one study may not be “many” or “most” across the five studies. This is particularly important because an experience is not judged important or not simply on the basis of whether it is a common experience or unique to that individual. | Thank you. We have rephrased sentences throughout the results section to better illustrate that we have described common experiences (common across participants within the same paper, but also common across different papers). |
| “Given that not many (especially not qualitative) studies were found that incorporated the  experiences of GBS from onset of symptoms and onwards” – this sentence is difficult to interpret – by using words such as Qualitative/focus groups etcetera in your search strategy you were only looking for QUALITATIVE studies. The fact that you retrieved some that weren’t qualitative isn’t a valid basis for making pronouncements about the quantitative studies which would not be representative of the ones you did not retrieve. Focus only on what you were studying, not what you accidentally retrieved along the way. | Thank you - we had not phrased this correctly. We have now deleted this sentence completely, which has also made the Discussion shorter as advised. |
| “well established guidelines (ENTREQ), which limited the potential for bias” You make the mistake here (and above in Methods) of assuming that following ENTREQ REPORTING guidelines somehow has an impact on the quality of the CONDUCT of your review. What reporting guidelines do is make it easier for a Reader to detect threats to bias (however that can be defined within a qualitative paradigm); they don’t make your review better. | Thank you. We have now changed the sentence to more accurately reflect what we were trying to say. The sentence now reads (p. 23): “The study followed a rigorous pre-specified protocol (registered with PROSPERO), which ensured that the review process was transparent and replicable.” |
| “A potential limitation of this review was only five studies met the inclusion criteria for meta- synthesis” – this is largely an irrelevancy – you should consider the adequacy of your data (cp GRADE CERQual on adequacy) which is a function of both numbers of studies and their richness. So reframe as adequacy – they could still be adequate even if not plentiful. If, however they are neither rich nor plentiful then you should specifically identify what data were missing.  Similarly the reason why there are no guidelines on how many studies should be included is that this is bringing in a quantitative criterion to a qualitative phenomenon. Why are MORE studies needed? Not because the numbers aren’t sufficient – if you are going to recommend more studies then you must state which perspectives or elements of the experience are missing and therefore need more data. Otherwise you are simply advocating wasted research monies! | Thank you- we have decided to remove this section from the limitations of the study and add a sentence under the strengths of the review to highlight the richness of the data we identified (p. 23): “Only five studies met the inclusion criteria for meta-synthesis; all studies included rich data, particularly numerous quotes, which effectively presented the participants’ lived experiences of GBS.” |
| “Including exclusively English-language papers could be considered a weakness, as important evidence may have been excluded due to language restrictions” This is true but you might wish to rebut this by saying “However, methods for translating concepts across languages, in addition to the initial challenge of translating them across studies, have not been sufficiently developed”. | Thank you, we have followed your advice and added your sentence (p. 24). If you would be happy with this, we would like to reference this as a personal communication with you. |
| There are several mentions of the role of “charities” in the Discussion. Given the funding for this study it would be good for the Reflexivity statement (as recommended above) to specifically address this. E.g. “The authors consider provision of information and support from charities as an important resource for patients, particularly with rare conditions, but such support may be available from multiple sources, and our view on potential sources may have been influenced by the funding for this study”. | Thank you, we have tried to address this in our “Reflexive Statement”. Although we may have been partly influenced by knowing that such a charity exists and how it can help patients, the main reason we have mentioned this in the Discussion is because the papers had included quotes from participants (which we had also included in the Results section) who mentioned that various GBS charities were useful (and sometimes the only) sources of both informational and emotional support for them. |
