# Supplementary material for: Patients’ experiences and perceptions of Guillain-Barré syndrome: A systematic review and meta-synthesis of qualitative research
Source: PLoS One. 2021 Feb 3;16(2):e0245826. doi: 10.1371/journal.pone.0245826 (PMC7857557; doi:10.1371/journal.pone.0245826)
Supplement: Attachment — Submitted filename: Response to reviewers 16.12.20.docx [file pone.0245826.s004.docx]

| Editor/reviewer comments | Response |
| --- | --- |
| Additional Editor Comments (if provided): Thank you for addressing the reviewers' comments the review is well written and addresses a significant area of research. Please note the recommendation below to further condense the article. I would recommend this for the introduction and adding in the recommendations for policy and practice within the discussion. | Thank you. We have condensed the text considerably and added recommendations for policy and practice in the discussion. |
| Reviewer #1: I thank authors for their efforts to improve the paper but from my point of view manuscript is still too long for publication as a journal article especially it important for intro and results sections. | Thank you. We have shortened the article as far as possible without changing the meaning of the text. The Background has been reduced from 1042 to 893 words and the Results from 2528 to 2103 words. |
